# Regulation of Gene Editing Activity Directed by Single-Stranded Oligonucleotides and CRISPR/Cas9 Systems

**DOI:** 10.1371/journal.pone.0129308

**Published:** 2015-06-08

**Authors:** Pawel Bialk, Natalia Rivera-Torres, Bryan Strouse, Eric B. Kmiec

**Affiliations:** 1 Department of Chemistry, Delaware State University, Dover, Delaware, United States of America; 2 Gene Editing Institute, Helen F. Graham Cancer Center, Newark, Delaware, United States of America; Osaka University, JAPAN

## Abstract

Single-stranded DNA oligonucleotides (ssODNs) can direct the repair of a single base mutation in human genes. While the regulation of this gene editing reaction has been partially elucidated, the low frequency with which repair occurs has hampered development toward clinical application. In this work a CRISPR/Cas9 complex is employed to induce double strand DNA breakage at specific sites surrounding the nucleotide designated for exchange. The result is a significant elevation in ssODN-directed gene repair, validated by a phenotypic readout. By analysing reaction parameters, we have uncovered restrictions on gene editing activity involving CRISPR/Cas9 complexes. First, ssODNs that hybridize to the non-transcribed strand direct a higher level of gene repair than those that hybridize to the transcribed strand. Second, cleavage must be proximal to the targeted mutant base to enable higher levels of gene editing. Third, DNA cleavage enables a higher level of gene editing activity as compared to single-stranded DNA nicks, created by modified Cas9 (Nickases). Fourth, we calculated the hybridization potential and free energy levels of ssODNs that are complementary to the guide RNA sequences of CRISPRs used in this study. We find a correlation between free energy potential and the capacity of single-stranded oligonucleotides to inhibit specific DNA cleavage activity, thereby indirectly reducing gene editing activity. Our data provide novel information that might be taken into consideration in the design and usage of CRISPR/Cas9 systems with ssODNs for gene editing.

## Introduction

The reengineering of mammalian genomes is a powerful genetic approach to both understanding gene function and developing new therapies for inherited diseases. While the traditional endpoint for genetic engineering has been to disrupt or disable a gene through complete knockout, it is now possible to direct single nucleotide exchange in an effective and efficient manner. Gene or genome editing can be catalyzed by a series of molecular tools that when used in various combinations accurately change the sequence of the DNA in a site-specific fashion.

Single-stranded DNA oligonucleotides (ssODNs) have been used for many years to engineer nucleotide changes in the genomes of many organisms [1–3]. And, while the mechanism of action and the regulatory circuitry [4] are being elucidated, at least in part, the low efficiency with which single-stranded oligonucleotides work in isolation has long hampered their development for therapeutic application. As a search for adjuvants that can enhance the frequency of single-stranded ODN–directed gene editing has proceeded, it was determined that the double-stranded DNA breaks induced prior to the introduction of the single-stranded ODN elevated the overall activity of gene editing between 5 to 10 fold [5–8]. Much of this dsDNA breakage was executed by the inherent activity of anticancer drugs, such as camptothecin, VP 16, VM 26 etc. While effective in promoting nucleotide exchange, these agents do not act at specific sites so that multiple non-discriminate cleavage events are likely to be taking place across the genome. Such random mutagenesis has the potential to lead to improper or inaccurate gene function or chromosomal activity. Thus, there was a need to identify enzymes or reagents whose double-stranded DNA cleavage activity could be programmed or at least partially controlled to act specifically and enhance gene editing.

Programmable nucleases, known to catalyze site-specific mutagenesis albeit with varying levels of off-site activity have been identified. Zinc finger nucleases [9] and Meganuclease [10] were among the first proteins to be used in such fashion. Recently, transcription activator–like effector nucleases (TALENs) have been found to cleave at site-specific locations in the genome of many organisms [11]. TALENs have also been used in combination with single-stranded ODNs to carry out gene editing activity, some of these studies have led to single nucleotide exchanges at specific sites in a functional gene [12–14]. We have used TALENs in combination with ssODNs to repair a point mutation in a single copy mutant eGFP gene where correction leads to functional genetic and biochemical readouts that can be assessed easily at both the genotypic and phenotypic levels [15,16]. TALENs reduce the amount of ssODNs needed to execute gene editing, most likely by providing a specific entry site for the oligonucleotide to invade the helix near the mutation and act as the donor DNA to repair the mutated base.

While TALENs have been quite effective in catalyzing this reaction, the design, construction and effective expression of these molecules in mammalian cells can be challenging. This ease-of-use issue has enabled the rapid development and acceptance of a new technology, a new tool for gene editing. Clustered, regularly interspace, short palindromic repeats (CRISPR) molecules associated with Cas9 endonucleases (CRISPR/Cas9), which function normally in the adaptive immunity pathway in some bacteria, have emerged as the preferred agent to catalyze site-specific DNA cleavage in many systems [17–22]. A major advantage in using CRISPR/Cas9 for genome editing is the simplicity with which the vectors expressing the CRISPR components can be created and utilized. While there are some notable sequence restrictions, i.e. the proto-spacer adjacent motif (PAM), by and large, these RNA- guided engineered nucleases (RGENs) have become main stream in this field. We decided to design and construct several RGENs aimed at correcting the mutant eGFP gene in our HCT 116–19 mammalian cell system [23]. Specifically, we wanted to evaluate CRISPR/Cas9-driven activity at the same genetic sites that had been successfully edited or repaired by the combinatorial approach of TALENs and ssODNs in the same system [15]. We built five RGENs guided by the location of the PAM sequence requirements within the mutant eGFP gene and carried out gene editing reactions with single-stranded oligonucleotides of 72 nucleotides in length. In some cases, RGENs were found to have higher editing activity than TALENs at several specific sites but both appear to be influenced by similar reaction parameters; strand bias, the requirement for proximal cut sites and enhanced response to cell cycle synchronization were found to be common in both nuclease complexes in a mutant gene. In addition, we generally find that ds breaks support gene editing at levels higher than single stranded nicks at the same sites.

## Material and Methods

### Cell Line and Culture Conditions

HCT116 cells were acquired from ATCC (American Type Cell Culture, Manassas, VA). The HCT116-19 was created by integrating a pEGFP-N3 vector (Clontech, Palo Alto, CA) containing a mutated eGFP gene. The mutated eGFP gene has a nonsense mutation at position +67 resulting in a nonfunctional eGFP protein [24]. For these experiments, HCT116 (-19) cells were cultured in McCoy’s 5A Modified medium (Thermo Scientific, Pittsburgh, PA) supplemented with 10% fetal bovine serum, 2mM L-Glutamine, and 1% Penicillin/Streptomycin. Cells were maintained at 37°C and 5% CO_2_. Custom designed oligonucleotides, 72NT, 72T and 72NT PM were synthesized from IDT (Integrated DNA Technologies, Coralville, IA).

### CRISPR Design and Construction

The mutant eGFP gene sequence was entered into the Zhang Lab’s online generator (http://crispr.mit.edu/) and the five CRISPR guide sequences which bind upstream and downstream with close proximity to target (TAG = 0) were chosen. The CRISPRs were constructed using standard cloning methods following the latest oligo annealing and backbone cloning protocol with single-step digestion-ligation[18]. The five CRISPR guide sequences were cloned into the pX330 backbone vector (Addgene plasmid 42230), a human codon-optimized SpCas9 and chimeric guide RNA expression plasmid, as well as into pX460 backbone vector (Addgene plasmid 48873) which is a D10A nickase mutant human codon-optimized SpCas9 and chimeric guide RNA expression plasmid. pX458 (Addgene plasmid 48138) was a gift from Feng Zhang and is a human codon optimized pSpCas9 and chimeric guide RNA expression plasmid with a 2A-eGFP. All plasmids were purchased through Addgene (https://www.addgene.org). Following construction, clones were verified by DNA sequencing by Genewiz Incorporated (South Plainfield, NJ).

### Transfection of HCT116-19 Cells and Experimental Approach

For experiments utilizing synchronized cells, HCT116-19 cells were seeded at 2.5 x 10^6^ cells in a 100mm dish and synchronized with 6μM aphidicolin for 24 hours prior to targeting. Cells were released for 4 hours prior to trypsinization and transfection by washing with PBS (-/-) and adding complete growth media. Synchronized and unsynchronized HCT116-19 cells were simultaneously transfected at a concentration of 5 x 10^5^ cells/100ul in 4mm gap cuvette (BioExpress, Kaysville, UT). Single-stranded oligonucleotides and CRISPR or Nickase plasmid constructs were electroporated (250V, LV, 13ms pulse length, 2 pulses, 1s interval) using a Bio-Rad Gene Pulser XCellTM Electroporation System (Bio-Rad Laboratories, Hercules, CA). Cells were then recovered in 6-well plates with complete growth media at 37°C for 48 hours prior to analysis.

### Analysis of Guide RNA and DNA Oligo Hybridization

Each guide RNA sequence and the 72NT oligo sequence were aligned and analyzed for base pairing and maximum ΔG values utilizing OligoAnalyzer 3.1 (https://www.idtdna.com/analyzer/Applications/OligoAnalyzer/). The ΔG is calculated by the longest stretch of complementary bases between the DNA and RNA structures the maximum ΔG value is determined as the free energy of the RNA sequence binding to its complement.

### Analysis of Gene Edited Cells and Transfection Efficiency

Fluorescence (eGFP^+^) was measured by a Guava EasyCyte 5HT Flow Cytometer (Millipore, Temecula, CA). Cells were harvested by trypsinization, washed once with 1x PBS (-/-) and resuspended in buffer (0.5% BSA, 2mM EDTA, 2μg/mL Propidium Iodide (PI) in PBS-/-). Propidium iodide was used to measure cell viability as such, viable cells stain negative for PI (uptake). Correction efficiency was calculated as the percentage of the total live eGFP positive cells over the total live cells in each sample. Error bars are produced from two sets of data points generated over two separate experiments using basic calculations of Standard Error. Statistical significance was performed by using two-sample unequal variance students T-test distribution to compare the value. *p<0.05

Sequence confirmation of ssODN/CRISPR edited cells was carried out by fluorescence-activated cell sorting of eGFP+ cells using a BD FACSAria II sorter- 488nm (100mw) (BD Biosciences, San Jose, CA). 1.35ug 72NT and 2ug CRISPR 2C transfected cells were sorted at 72 hours post electroporation. Immediately, DNA was isolated from each sample was using the DNeasy Blood and Tissue kit (Qiagen, Hilden, Germany). The targeted site was amplified via PCR using forward primer, 5’CTGGACGGCGACGTAAACGGC and reverse primer, 5’ ACCATGTGATCGCGCTTCTCG. PCR cleanup was performed using the QIAquick. PCR purification kit (Qiagen, Hilden, Germany) and the purified samples were sent for sequencing to Genewiz Incorporated (South Plainfield, NJ).

Unsynchronized HCT116-19 cells were harvested and electroporated at a concentration of 5x10^5^cells/100ul with 2ug of the indicated CRISPR/Cas9 (2C, 3C, 5C and empty pX458 vector) plus 1.35ug of either 72NT or 72 PM. Following electroporation, transfection efficiency is determined after 24 hours of incubation by the percentage of total viable eGFP+ cells in the population. The normalized correction efficiency was determined after 48 hours of incubation as the percentage of total viable eGFP+ cells in the population divided by the transfection efficiency.

### RFLP Analysis of CRISPR/Cas9 Cleavage Activity

HCT116-19 test samples were electroporated at a concentration of 5 x 10^5^ cells/100ul in 4mm gap cuvette (BioExpress, Kaysville, UT) with 2ug of CRISPR/Cas9 constructs 2C and 3C as well as 2ug 2C + 1.35ug 72NT PM and 2ug 3C + 1.35ug 72NT PM. Cells were then recovered in 6-well plates with complete growth media at 37°C for 72 hours. DNA was isolated using the Blood and Tissue DNeasy kit (Qiagen, Hilden, Germany). RFLP analysis was performed on 181bp amplicons that were created using forward primer, 5’GAGGGCGATGCCACCTACGGC and reverse primer, 5’GGACGTAGCCTTCGGGCATGGC. PCR samples were purified using the QIAquick PCR purification kit (Qiagen, Hilden, Germany) and treated with the AvrII restriction enzyme following the manufactures protocol. Digested samples were loaded along with NEB 2-log DNA ladder (NEB, Ipswich, MA) into a 2% TBE agarose gel for analysis. SYBR Gold (Invitrogen, Carlsbad, CA) was used to stain the gel and images were acquired by the Gel Doc EZ System (BioRad, Hercules, CA) to create an electrophoregram. Using Bio Rad’s Image Lab software, automated lane detection was performed, followed by selecting bands. Using the software, the concentration of each band represented by a peak on the electrophoregram was derived from the area of each peak as a percent of the total lane peak area.

### SURVEYOR Analysis of CRISPR/Cas9 Cleavage Activity

HCT116-19 cells were electroporated at a concentration of 5 x 10^5^ cells/100ul in 4mm gap cuvette (BioExpress, Kaysville, UT) with 2ug of each CRISPR/Cas9 construct. Cells were then recovered in 6-well plates with complete growth media at 37°C for 72 hours. Genomic DNA was extracted and purified using the DNeasy Blood and Tissue kit (Qiagen) following the manufacturer’s protocol. The genomic region surrounding the mutant eGFP gene locus targeted by each gRNA as well as an untreated sample was PCR amplified using Phusion High-Fidelity PCR Master Mix with HF Buffer (Thermo Scientific). 200ng of each PCR product was mixed with 200ng of PCR product from the untreated sample and subjected to a heteroduplex formation: 95°C for 10 minutes, 95°C to 85°C with a ramp rate of -2°C/s, 85°C for 1 minute to 75°C at-.1°C/s, 75°C for 1 minute to 65°C at-.1°C/s, 65°C for 1 minute to 55°C at-.1°C/s, 55°C for 1 minute to 45°C at-.1°C/s, 45°C for 1 minute to 35°C to 25°C at-.1°C/s, 25°C for 1 minute. After duplex formation products were treated with SURVEYOR Nuclease S and SURVEYOR Enhancer S (IDT Technologies) for 30 minutes at 42°C, gel electrophoresed and stained with SYBR Safe DNA stain (Life Technologies). Gels were imaged with a Gel Doc EZ Imager (Bio-Rad) and densitometry was performed by measuring the area under the curves of each band, using the Image Lab software (Bio-Rad). Calculations were based on the following formulas:

%cleaved=sum of cleaved products/sum of cleavage products+parent band

## Results

**Fig 1:**
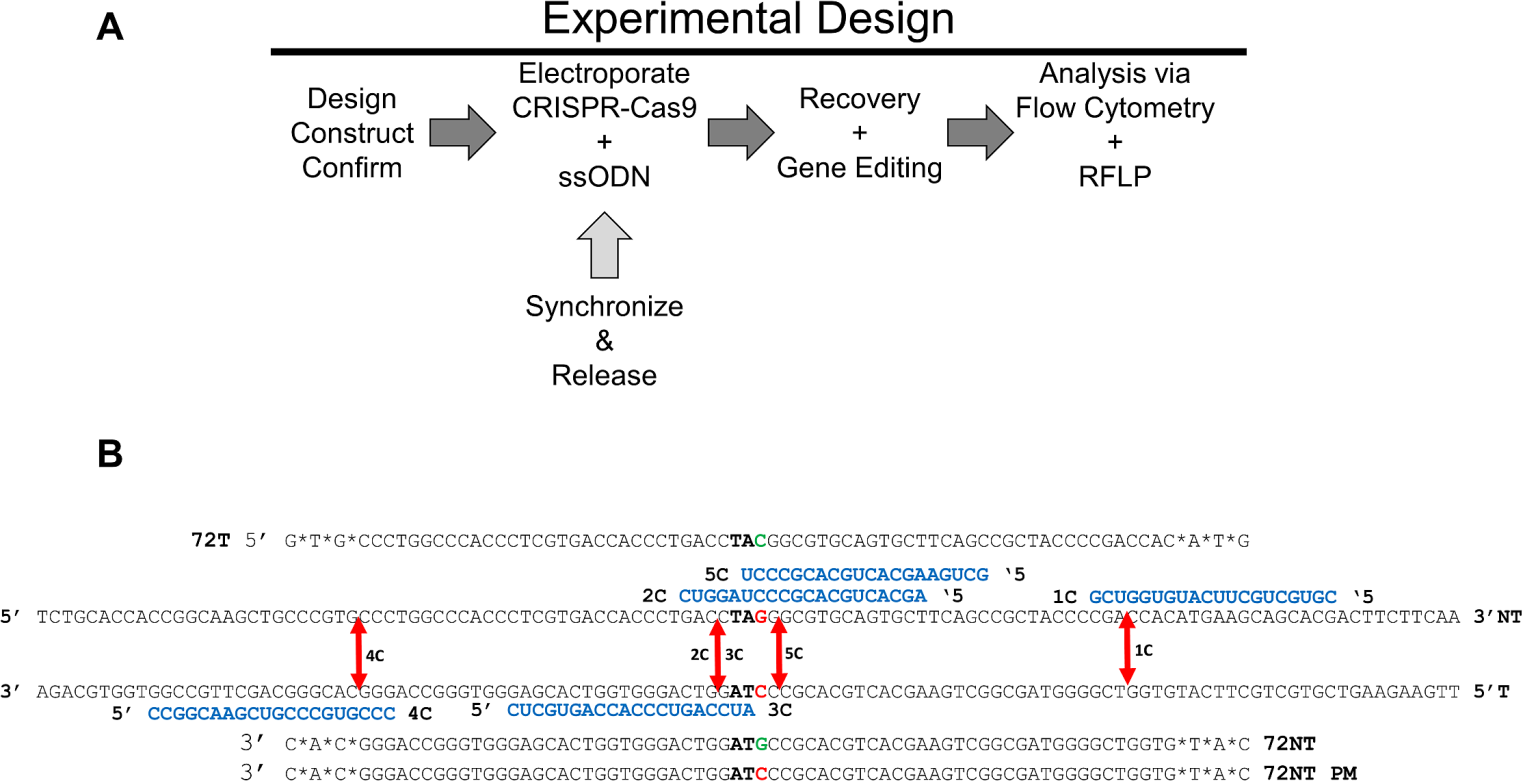
Experimental Design and Mutant eGFP Gene Editing System (A) CRISPR/Cas9 constructs were designed and built following published guidelines and protocols [42]. Unsynchronized or synchronized and released cells were transfected via electroporation with CRISPR/Cas9 construct and ssODNs and allowed to recover before analysis. Gene editing was assessed by flow cytometry and CRISPR/Cas9 activity was measured by RFLP using restriction enzyme AvrII. (B) A segment of the mutant eGFP gene is shown with the three ssODNs, 72NT, 72T and 72NT PM respectively aligned above or below their respective binding sites. Phosphorothioate modified and protected ends are denoted with *.The five arbitrarily named (1C-5C) custom CRISPR/Cas9s RNA guide sequences are depicted in blue with their predicted cleavage sites shown by the red arrows. The effected codon is shown in bold with the mutant base to be edited in red. The base driving the gene editing conversion is shown in green.

Fig 1A presents the experimental design for work outlined in this manuscript. The CRISPR and Nickase constructs and single-stranded oligonucleotides were designed to target the mutated eGFP gene in HCT 116 cells. The single copy integrated eGFP gene with a mutant TAG codon has been one of the most useful targets for gene editing experiments [23,24]. By the combined action of the CRISPRCas9/Nickase enzyme complex and the single-stranded oligonucleotide, the TAG codon can be converted to TAC, a tyrosine codon that enables the production of functional eGFP. This system enables a rapid readout at both the phenotypic (functional) and genotypic levels. HCT 116–19 cells were electroporated with a specific CRISPR /Cas9 construct or the Nickase construct and single-stranded ssODNs. In some cases, the cells were synchronized for 24 hours and then released for four hours prior to electroporation. After 48–72 hours of recovery, the cells were analysed by flow cytometry and by restriction enzyme digestion. The latter assay is used to confirm specific CRISPR/Cas9 cleavage activity at the target site.

**Fig 2:**
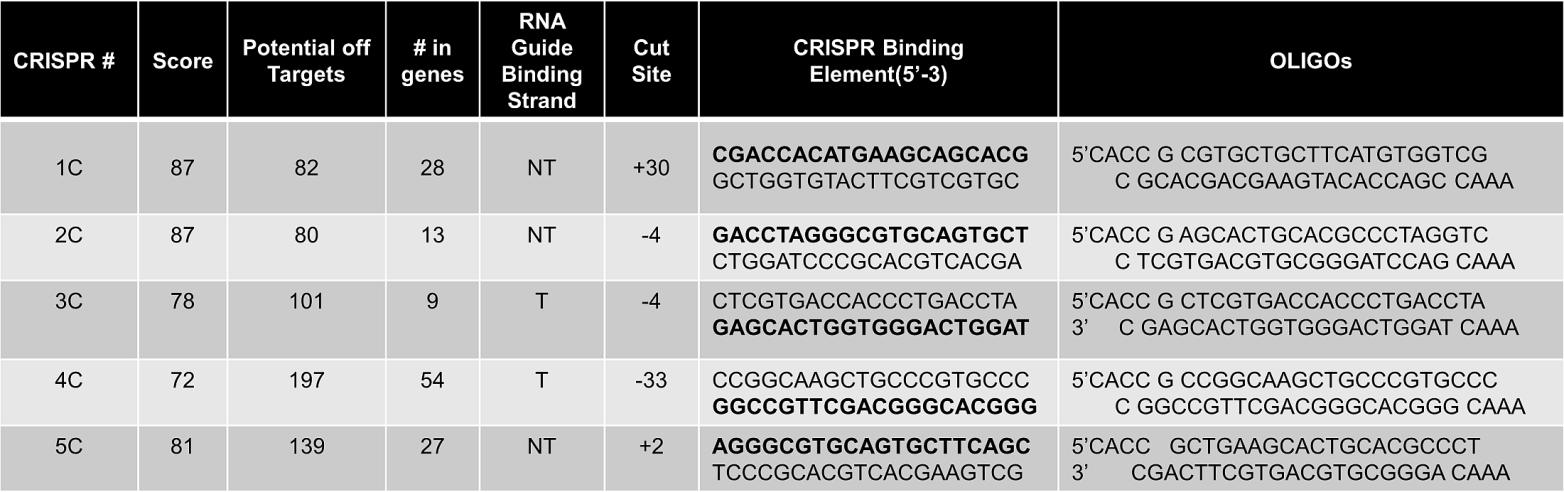
Summary of Constructed CRISPR/Cas9 gRNAs. Each designed guide RNA was generated from the crispr.mit.edu online algorithm. CRISPR # designates the gRNA name used in this manuscript. Score represents the likelihood of the gRNA binding and causing unwanted mutations (score of 100 is the best possible gRNA). Possible off-target effects is the total number of individual loci across the genome that could be cleaved with the number of those within in genes (exons) listed in the following column. RNA guide binding strand denotes which stand the guide RNA will target (NT = non-transcribed, T = transcribed). Cut site is where the gRNA will direct the DSB break to be made relative to TAG = 0. CRISPR binding element shows the segment of the eGFP gene that the guide will bind with the actual bound strand in bold. Oligos used for construction with correct linkages are shown in the final column.

**Fig 3:**
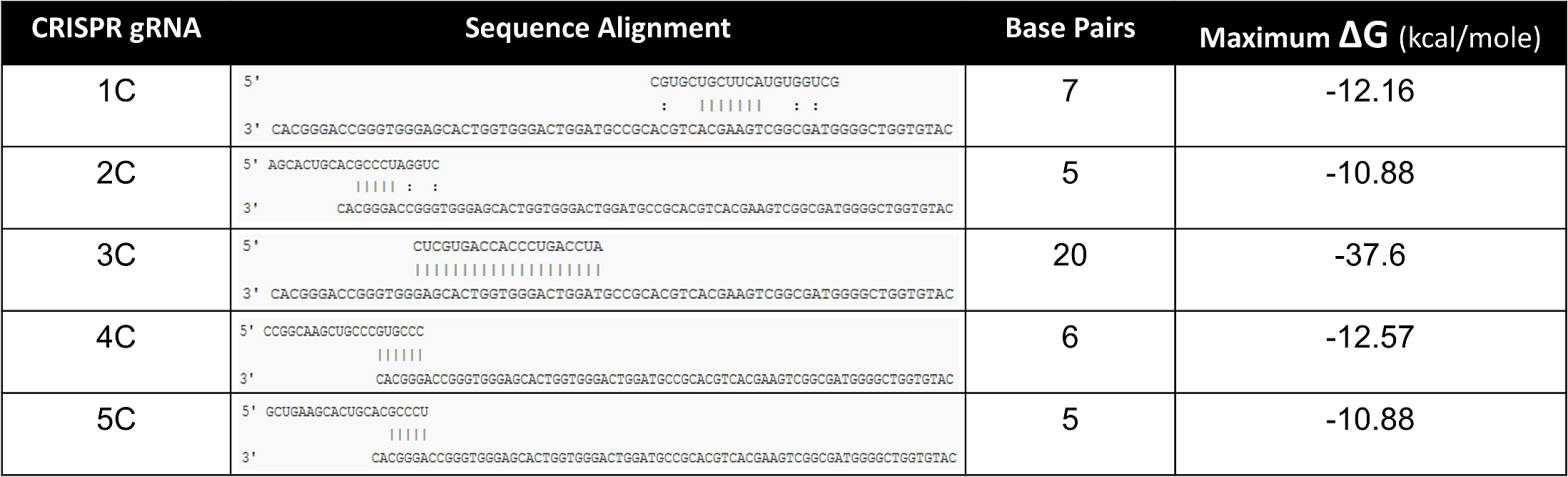
Free Energy Heterodimer Values for CRISPR Guide RNA and ssODN Combinations. Each gRNA sequence was aligned and analyzed for base pairing and maximum free energy (ΔG) values utilizing the IDT heterodimer calculator and measured in kcals/mole. The highest ΔG value for each gRNA/72NT ssODN pairing is shown. Solid lines represent the longest stretch of direct base pairing while dotted lines represent additional complimentary bases (not calculated in ΔG). A more negative ΔG value represents a stronger binding capacity.

The specific design and locations of all the CRISPR/Cas9 complex cleavage and the appropriate, specific single-stranded oligonucleotides used in targeting are presented in Fig 1B and Fig 2. Each arrow indicates the position of cleavage by the appropriate CRISPR/Cas9 with the RNA guide sequences depicted in blue. Above and below the target sequence are the single-stranded oligonucleotides used in this study; 72T, 72NT and 72NT-PM. The RNA guide sequence is also aligned above or below based on its complementarity to the gene, at the position of hybridization; the top strand of the gene is the non-coding strand (NT). For example, the ssODN (72NT) is complementary to the top (NT) strand and therefore complementary to a guide RNA sequence that hybridizes to the lower/bottom/T strand. 72T has the opposite polarity being complimentary to the bottom or T strand as depicted. As such, CRISPRs 2C and 3C cut at the same spot, but 3C contains a guide sequence complementary to 72NT to which it will anneal. 3C has a ΔG of hybridization of -37.6 kcal/mole and thus has a significant probability of becoming annealed whereas 2C and 5C have ΔGs of -10 kcal/mole (Fig 3). 4C has a ΔG of approximately -12 kcal/mole because there are only six bases in common whereas the guide sequence in CRISPR 3C is completely complementary. These alignments and free energy measurements could be quite informative since complimentary guide sequences are able to hybridize to the single-stranded oligonucleotide with opposite polarity, but bearing complementarity. 5C has a low ΔG, with a guide on the NT strand and is downstream of the target base. Previous data suggest, cleavage 5’ (upstream) relative to the target nucleotide (here the G of TAG) produces the highest enhancement of gene editing activity [15]. A double-stranded break 3’ to the target base however has been shown to reduce the level of gene editing. We acknowledge that re-cutting by 3C could be occurring and such activity could lead to a reduction in productive gene editing levels given the cut sites for 2C and 5C are within the seed sequence of the target, it is unlikely that reoccurring endonuclease cleavage takes place.

**Fig 4:**
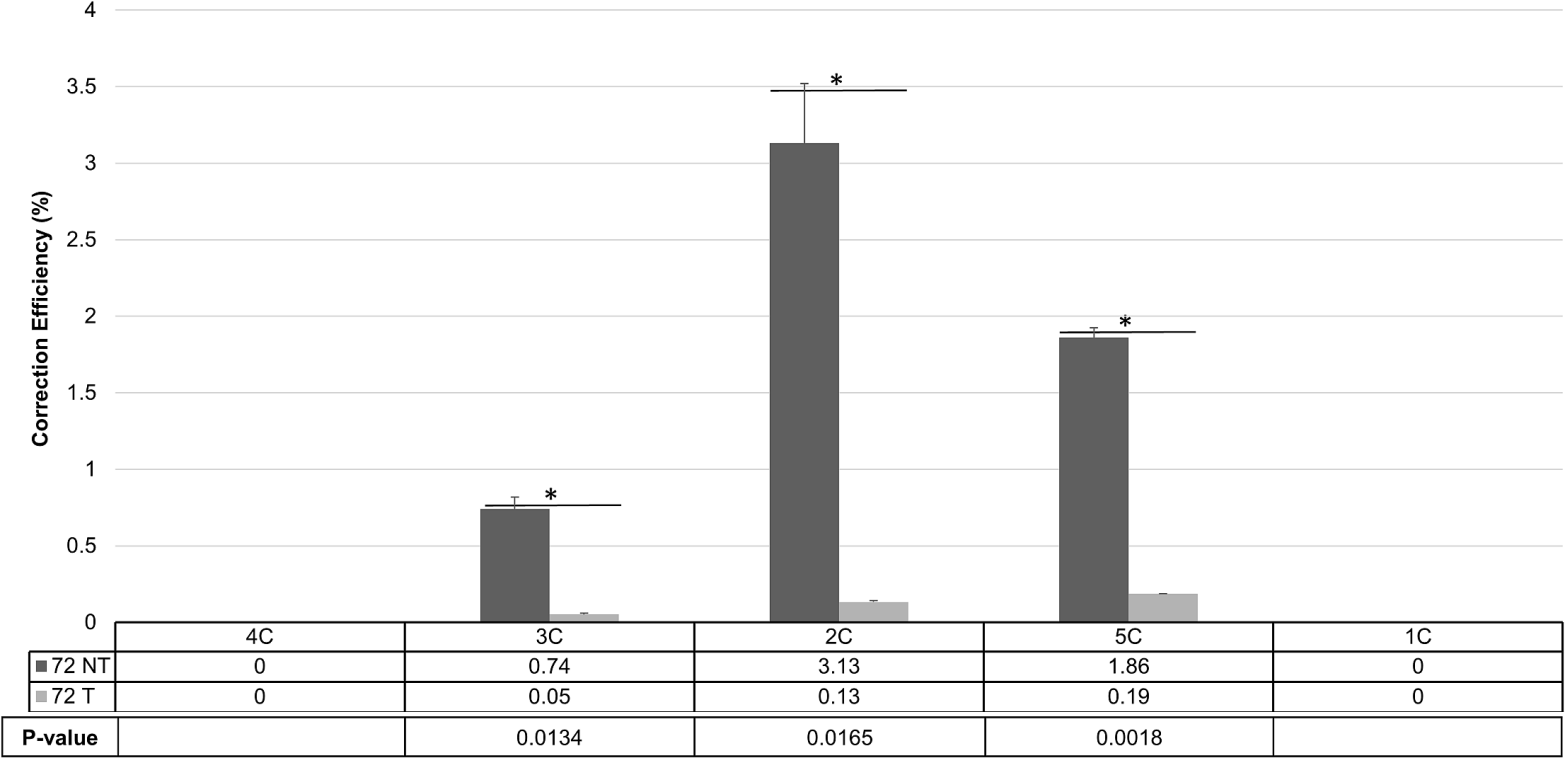
CRISPR/Cas9 and ssODN Gene Editing Activity. Unsynchronized HCT116-19 cells were harvested and electroporated at a concentration of 5x10^5^cells/100ul with 2ug of the indicated CRISPR/Cas9 plus 1.35ug of either 72NT or 72T. Following electroporation, cells were allowed to incubate for 48 hours and correction efficiency was determined by the percentage of total viable eGFP+ cells in the population. 1C– 5C CRISPR/Cas9 complexes and are listed left to right relative to their cut site. The numbers below indicate the average correction efficiency. Error bars represent standard error.

Single-stranded oligonucleotides were mixed with the appropriate CRISPR/Cas9 complex and allowed to carry out gene editing for 48 hours. After this time, correction of the mutant eGFP gene was measured by FACS. The results are presented in Fig 4. On the left side bar graphs, the single-stranded oligonucleotide that hybridizes to the non-transcribed strand, 72 nucleotides in length (72NT), was used in combination with each individual CRISPR/Cas9 complexes. Gene editing levels vary widely among the five complexes used in the experiment. CRISPR 2C and 5C promote maximum activity primarily because they both cleave proximal to the targeted G nucleotide. 5C cleaves one base 3’ to the targeted base and 2C cleaves 3 bases upstream. CRISPR/Cas9 3C anneals on the opposite strand, the transcribed strand of the target gene, albeit at the same site as 2C, which anneals to the NT strand. On the right side of the graph, the single-stranded oligonucleotide, 72T that hybridizes to the transcribed strand of the gene was used in combination with the same CRISPR/Cas9 complexes used in the previous set. As expected, and consistent with previous data [3,15,25–32] the 72T enables a lower level of gene editing activity in this case, independent of the type of CRISPR/Cas9 complex used. Once again, complexes that cleave near the targeted nucleotide exhibit the highest level of gene editing activity. CRISPR 4C and 1C, which target distal to the targeted base, exhibit, in both panels, much lower levels of gene editing activity independent of which ssODN is used.

Gene editing, driven by single-stranded oligonucleotides and TALENs, has been previously shown to be enhanced when the targeted cells are synchronized at the G1/S border and released for four hours prior to the introduction of the editing tools [1,6,25,26,33,34]. Recently, Lin et al (2014) [35] blocked the cell cycle using aphidicolin in an analysis of CRISPR driven genome editing. This perturbation reduced HDR frequency dramatically supporting the notion that cells transiting S-phase are more amenable to gene editing activity, aligning with our own and others’ observations cited above. To examine the effect of cell synchronization and release on CRISPR/Cas9 targeting, driven by single-stranded ODNs, cells were assembled at the G1/S border for 24 hours and then released for four hours prior to the introduction of CRISPR/Cas9 complexes 2C, 3C and 5C respectively. Inclusion of 72NT completed the reaction mixture and gene editing efficiency was measured 48 hours post electroporation. The results, shown in Table 1, support the notion that gene editing frequencies can be elevated in reactions containing CRISPR/Cas9 complexes and ssODNs if cells are synchronized and released cells before the addition of the gene editing machinery.

**Table 1:** S-phase Increases CRISPR/Cas9 and ssODN Directed Editing.

**CRISPR /Cas9**	**Unsynchronized cells CE (%) ± SD**	**Synchronized cells CE (%) ± SD**	**P-value**
2C	3.13 ± 0.55	4.59 ± 1.39	0.3013
3C	0.74 ± 0.11	0.92 ± 0.03	0.1552
5C	1.87± 0.10	5.62 ± 0.23	*0.0019

HCT116-19 cells were seeded at 2.5x10^6^ cells in a 100mm dish and synchronized for 24 hours with 6uM aphidicolin then released for 4 hours. Synchronized and unsynchronized cells were electroporated at a concentration of 5x10^5^ cells/100ul with CRISPR/Cas9 and 72NT ssODN under the standard reaction conditions. Following electroporation, cells were seeded in 6-well plates and allowed to recover for 48 hours before flow cytometry analysis was carried out. Correction efficiency (%) was determined by the number of viable eGFP+ cells. Each sample set was performed in duplicate and ± represent calculated standard deviation per sample. The unsynchronized data is the same shown in Fig 4. Statistical analysis was performed using two-sample unequal variance students T-test distribution to compare the value of correction efficiency between synchronized and un-synchronized cells when treated with CRISPR/Cas9.

*p<0.05

**Fig 5:**
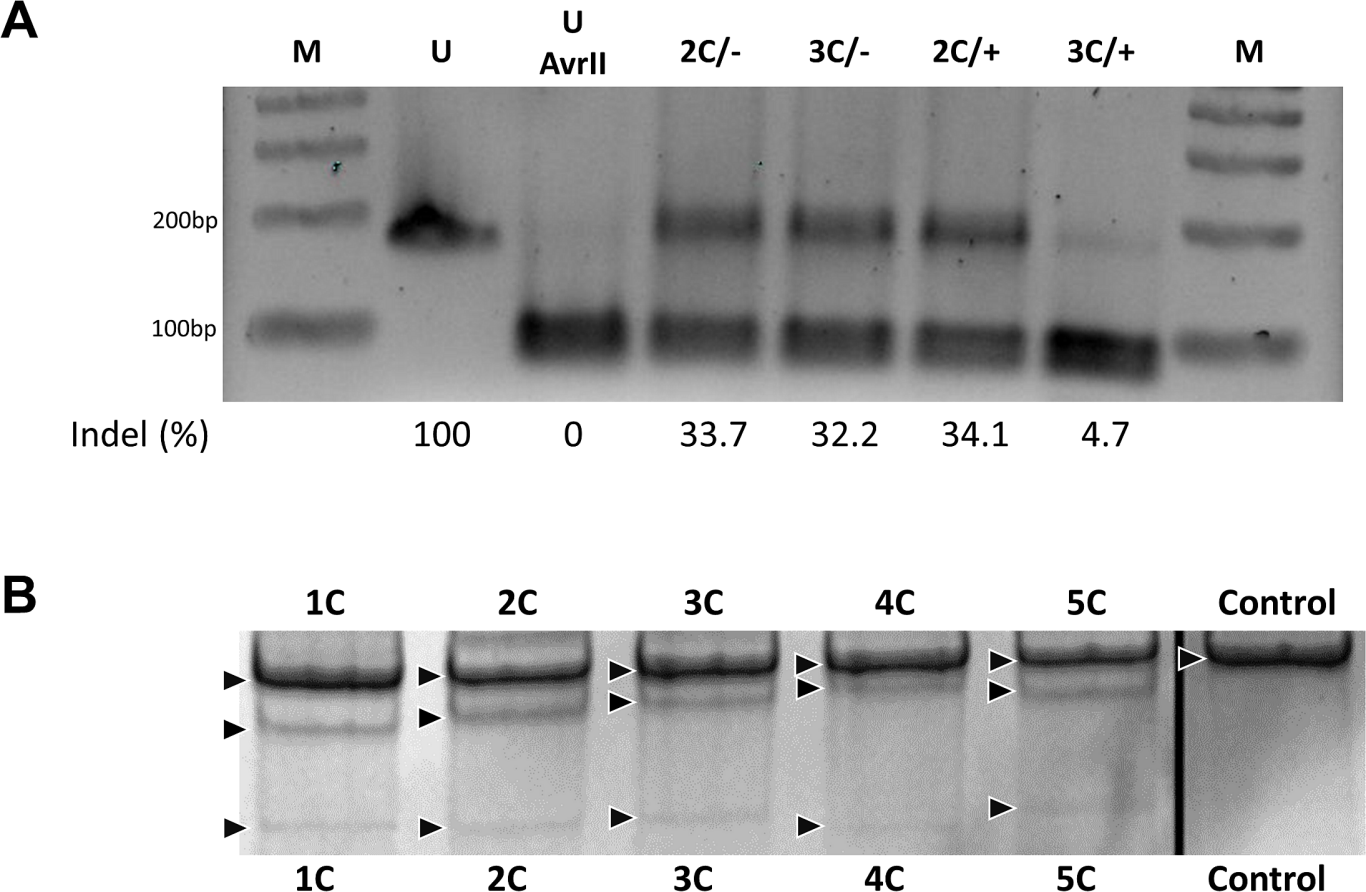
CRISPR/Cas9 Cleavage Activity. (A) 2% TBE agarose gel analysis of cleavage products generated by 2C and 3C CRISPR/Cas9 complexes at the eGFP gene target site. AvrII restriction enzyme was used to digest the amplified region of the eGFP gene. 2C/-, CRISPR 2C with no ssODN; 2C/+, CRISPR 2C with 72NT etc. Untreated 181bp (U) and Untreated + AvrII 181bp PCR products were used as internal controls with AvrII digested 2C, 3C and 2C + 72NT PM and 3C 72NT PM 181bp PCR samples. Densitometry was performed on all samples and percent cleavage (181bp band) is indicated below each sample. (B) SURVEYOR assay comparing the cleavage efficacy of each gRNA as the percent of indel formation. Arrowheads indicate parental bands and cleaved products.

The results presented in Fig 4 indicate that the CRISPR/Cas9 complex, 3C, catalyses gene editing events at a 4 to 5 fold lower frequency than its counterpart CRISPR/Cas9 complex 2C. Since both 2C and 3C cleave the target site at the same position, the difference in activity profiles is intriguing. As indicated above, the guide sequence of 2C anneals to the non-transcribed strand of the eGFP gene. In contrast the guide sequence of 3C anneals to the non-transcribed strand as shown in Fig 1B. Interestingly, 72 NT is complementary to the guide sequence of 3C but is not complimentary and has the same polarity as the guide sequence of 2C. Thus in agreement with the data presented in Fig 3 where the maximum Δ G of 3C (and the single-stranded ODN, 72 NT) is -37.6, it is likely that the reduction in gene editing activity exhibited by 3C is due to hybridization of the guide sequence of 3C with 72 NT. We tested this prediction directly by carrying out reactions that included 2C and 3C in the presence or absence of a perfect match single-stranded oligonucleotide complementary to the non-transcribed strand (72 NT–PM). 72 NT–PM is identical to 72 NT except it does not create the mismatch at the target base, it is perfectly complementary to the nucleotide sequence of the non-transcribed strand. This perfectly matched oligonucleotide needs to be used because the activity of 72 NT will modify the site so the restriction enzyme AvrII will not recognize the site thereby invalidating the assay. After 72 hours of reaction time, genomic DNA was isolated and a 181 base pair fragment, spanning the target site, was amplified by PCR. The amplified fragment was digested by the restriction enzyme AvrII which cleaves at the target region and thus can be used as an indicator of double strand DNA cleavage activity [15]. The resultant mixture of DNA fragments were electrophoresed through agarose and the results are presented in Fig 5A. The uncut fragment migrates to a position consistent with its size of 181 bases whereas AvrII- treated DNA is cleaved to completion (90 and 91 bases, respectively). In reactions containing either 2C or 3C alone (in the absence of the single-stranded DNA oligonucleotide, 72NT) the results clearly show high levels of CRISPR/Cas9 cleavage activity. Densitometry tracings place the level of resistant bands at 33% for 2C and 32% for 3C respectively. When the oligonucleotide 72 NT–PM is included in the reaction mixture with 2C, the level of cleavage activity remains identical to reaction mixtures that included only 2C. In contrast, a barely detectable level of a resistant band is seen in reaction mixtures which contain 3C and 72 NT–PM. These results are consistent with the notion that 72 NT, the single-stranded oligonucleotide used in the gene editing reactions presented in Fig 4, disables the action of CRISPR/Cas9 complex 3C presumably by hybridizing to the guide RNA. Since the guide RNA of CRISPR/Cas9 complex 2C is identical, not complementary to 72 NT (same polarity), the oligonucleotide is unable to titrate the guide sequence of CRISPR/Cas9 2C. Thus, 2C remains active in promoting gene high levels of editing activity as seen in Fig 4. We also utilized the SURVEYOR cleavage assay [36] to confirm CRISPR/Cas9 activity (Fig 5B). Each of the constructs used in this study was found to be sufficiently active in promoting DNA breakage. Our intent here is not to develop correlations between the level of gene editing and DNA cleavage since this particular assay is sensitive to some variability and activity can be dependent on background signals when polymorphisms are present in the target genome [37]. We have experienced that variability in our own hands. Rather, we use the SURVEYOR assay simply to support our other indicators of the CRISPR/Cas9 general cleavage activity of our expression constructs.

**Fig 6:**
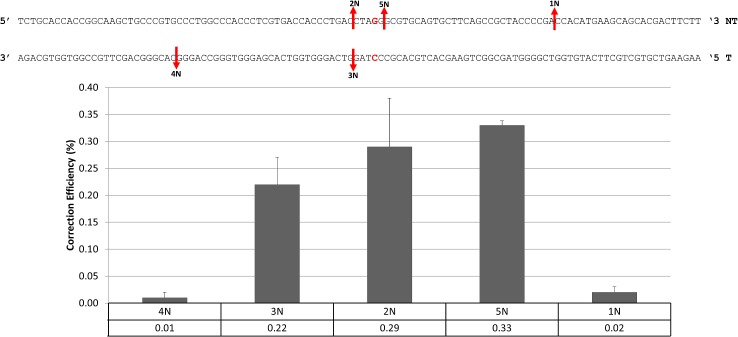
Gene Editing Activity with CRISPR/Cas9 Nickases and ssODNs. Unsynchronized HCT116-19 cells were electroporated at a concentration of 5x10^5^ cells/100ul with 2ug of the indicated CRISPR/Cas9 Nickase (1N, 2N, 3N, 4N, 5N) plus 1.35ug of 72NT. Following electroporation, cells were allowed to incubate for 48 hours. Correction efficiency was determined by the percentage of total viable eGFP+ cells in the population as described previously. Each treatment was performed in duplicate and error bars represent standard error.

While CRISPR/Cas9 complexes can induce efficient cleavage and promote enhanced levels of gene editing, directed by single-stranded oligonucleotides, concern has been raised that intact CRISPR/Cas9 molecules promote high levels of off-site mutagenesis. Hence, a significant amount of effort has been put into constructing a variation on the CRISPR/Cas9 theme; single Nickase enzymes that cleave only one strand supposedly reduce off-site mutagenesis [18,38]. We decided to test Nickase activity in the catalysis of gene editing on the mutant eGFP gene using complexes consisting of part of the CRISPR/Cas9 complexes depicted and described in Fig 1B. In the top panel of Fig 6, the red arrows indicate the position of an individual Nickase (N) cleavage. For example, 4N cleaves upstream from the target base but on the transcribed strand whereas Nickase 3N cleaves the transcribed strand as well, but only a few bases upstream from the target base. 2N, 5N and 1N cleave the non-transcribed strand alone at the indicated positions. When each of these Nickase complexes were tested with the single-stranded oligonucleotide, 72 NT and the level of correction monitored by FACS, we once again see that cleavage proximal to the target site provides the highest level of gene editing activity. Cleavage at distal sites promotes lower activity. The overall level of gene editing, however, is 10 to 15 fold lower than when the complete (corresponding) CRISPR/Cas9 complex, which cleaves both strands of the DNA, is used. Thus, while off-site mutagenesis may be avoided or diminished, the level of gene editing using single-stranded oligonucleotides is greatly diminished when a single-strand cut on only one of the two strands of the DNA is made by the appropriate Nickase complex. In addition, because the levels again are very low, we have to be aware of a possibility that the same processes could occur and outcomes achieved in both types of reactions although differing in enzymatic activity.

**Fig 7:**
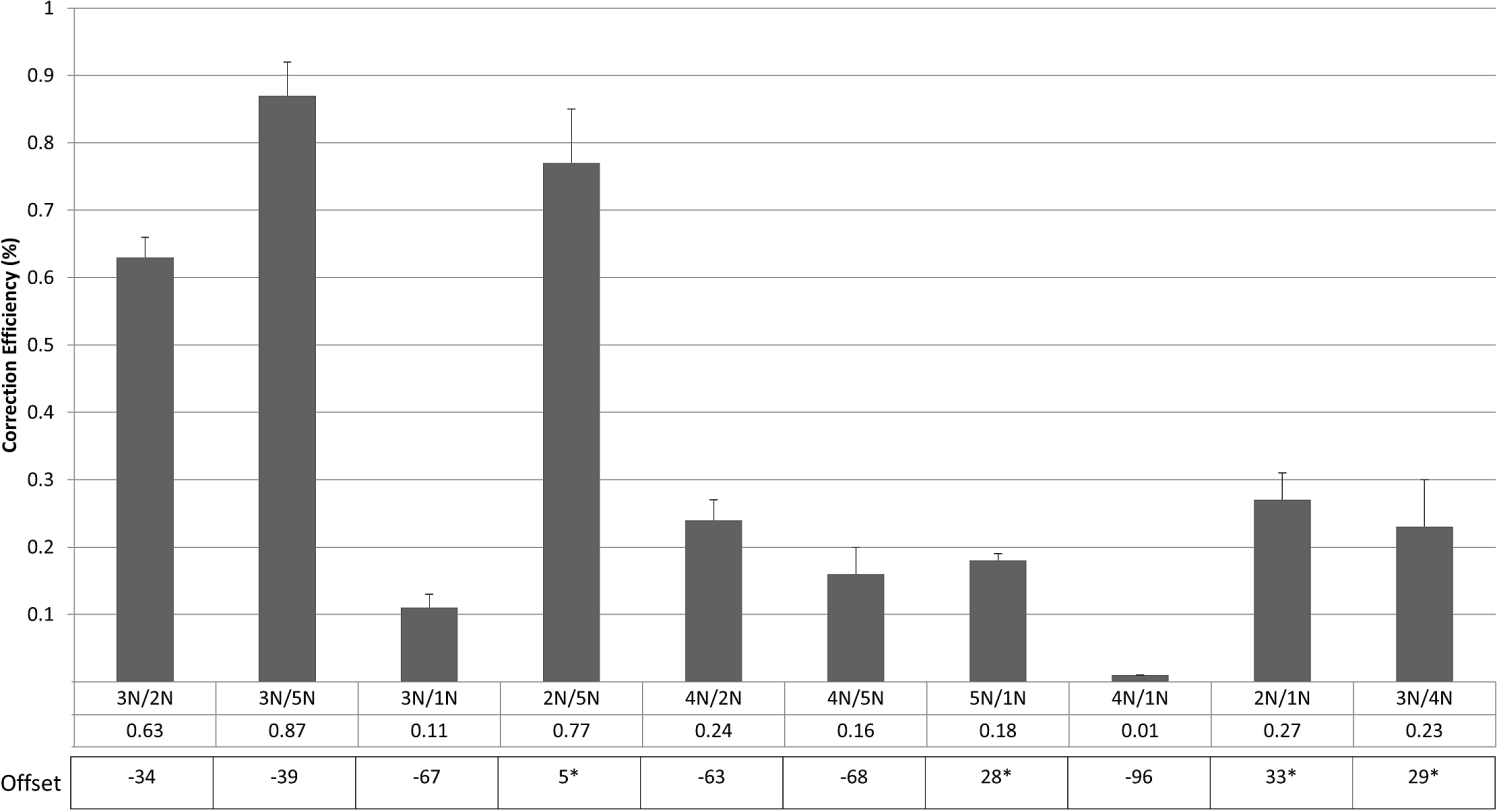
Double Nicking Nuclease Array of Gene Editing. Unsynchronized HCT116-19 cells were electroporated with 1ug of each of the indicated combinations of CRISPR/Cas9 nickases (1N, 2N, 3N, 4N, 5N) plus 1.35ug of 72NT. Offsets denoted with a star (*) represent nicking pairs which induce nicks on the same strand. Following electroporation, cells were allowed to incubate for 48 hours. Correction efficiency was determined by the percentage of total viable eGFP+ cells in the population as described previously. Each treatment was performed in duplicate and error bars represent standard error.

Reconstitution of the double strand break via a combination of Nickase activities does not resurrect the same level of gene editing as produced by the intact CRISPR/Cas9 complexes. Most of these combinations seen in Fig 7, including 3N/5N, produce efficient gene editing reactions so that a pure double strand break catalyzed by a CRISPR/Cas9 complex, at the site, or 5’ to the approximate location of the target base is necessary for efficient gene editing. It is possible, as described above; some of the combinations may not work because 3N and 4N have guide sequences that can be titrated by the single-stranded oligonucleotide, rendering them non-functional, while synergistic activity is observed when 2N and 5N are used (see Discussion).

**Fig 8:**
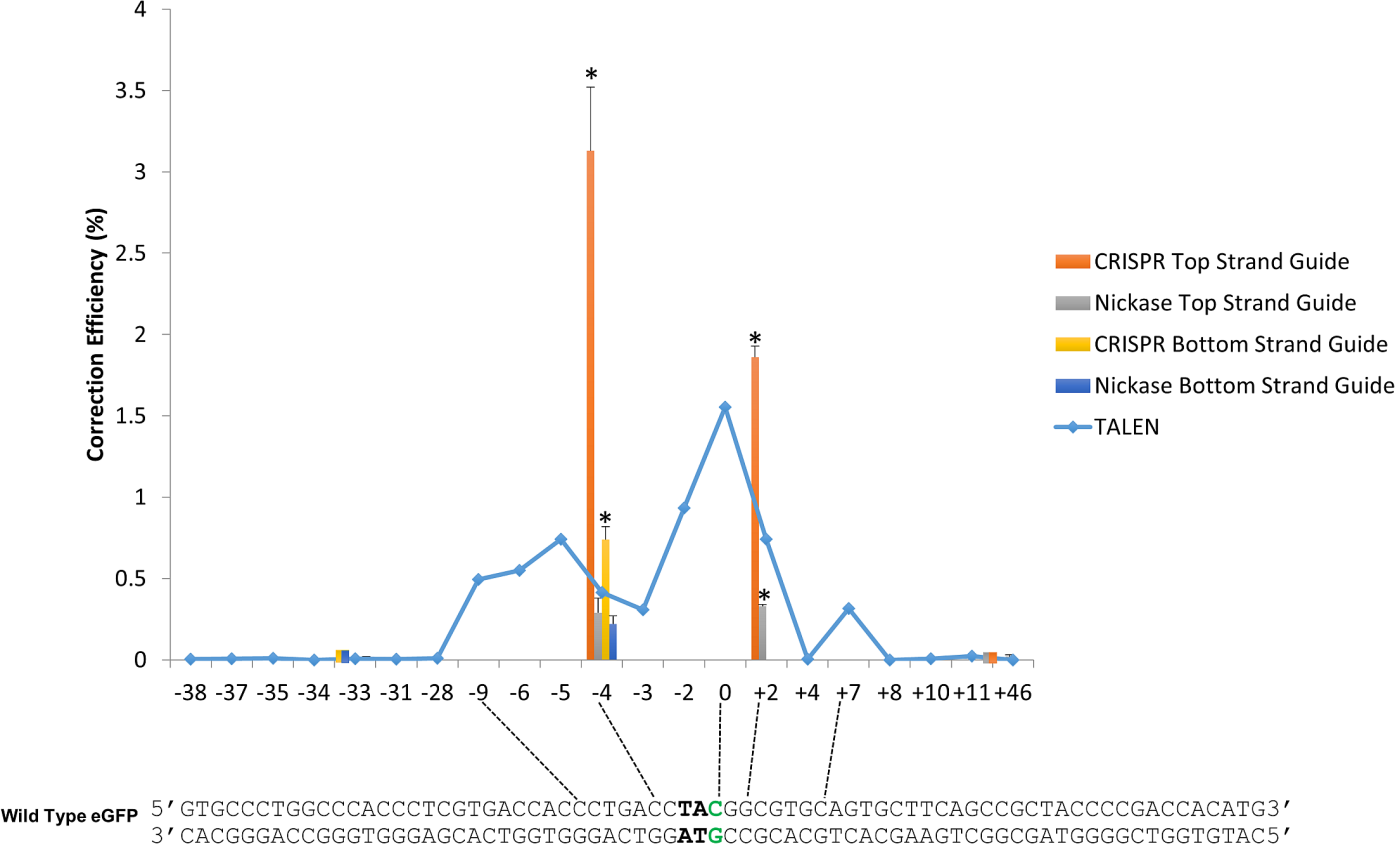
Activity Profile of Gene Editing catalyzed by TALENs, CRISPRs or Nickases at the target eGFP gene. **ssODN directed gene editing activity utilizing TALENs, CRISPR/Cas9s or CRISRP/Cas9 nickases was compiled and plotted within the region of the target eGFP gene.** Cytosine of the corrected tyrosine codon is designated as base 0. TALEN data was derived from previous work [15]. tatistical analysis was performed using two-sample unequal variance students T-test distribution. *p<0.05 (see Table 2).

In a previous publication, we defined the boundaries of efficient gene editing directed by TALENs and single-stranded oligonucleotides [15] as a region spanning the target nucleotide. We found that moderate gene editing (approximately 1%) diminishes upstream when the distance is expanded from -8 /-9 to -28 respectively. The distance downstream appears to be more restricted with editing levels reduced between +6\+7 and +8\+10 [15]. ur data set is admittedly low, but we do observe significant levels of gene editing at position -4 (upstream) while no activity is seen at position -33 (upstream). The data generated in the present work is overlaid with the previous TALEN/single-stranded ODN data (Fig 8) while statistical analysis is presented as Table 2. In direct comparison, CRISPR/Cas9 complexes that act at proximal regions also approximate the highest level of activity as the levels promoted by the TALEN and single-stranded oligonucleotide combination that act in the same region. Both technologies support the notion that creating a double strand break near the targeted nucleotide, destined for change, promotes the highest level of gene editing activity.

**Table 2:** Comparing Activity of Gene Editing catalyzed by TALENs, CRISPRs or Nickases at the target eGFP gene.

**TALEN**	**CRISPR/Nickase**	**P-value (0.05)**
****-4****	3C	*0.0198
****-4****	2C	*0.0517
****-4****	3N	0.2941
****-4****	2N	0.0628
****-4****	5C	*0.0106
****-4****	5N	*0.0473

Significance was determined using a T-test to compare the TALEN value to the CRISPR/Nickase value for each set presented in the table. A * was marked on graph to show those that were statistically significant.

**Fig 9:**
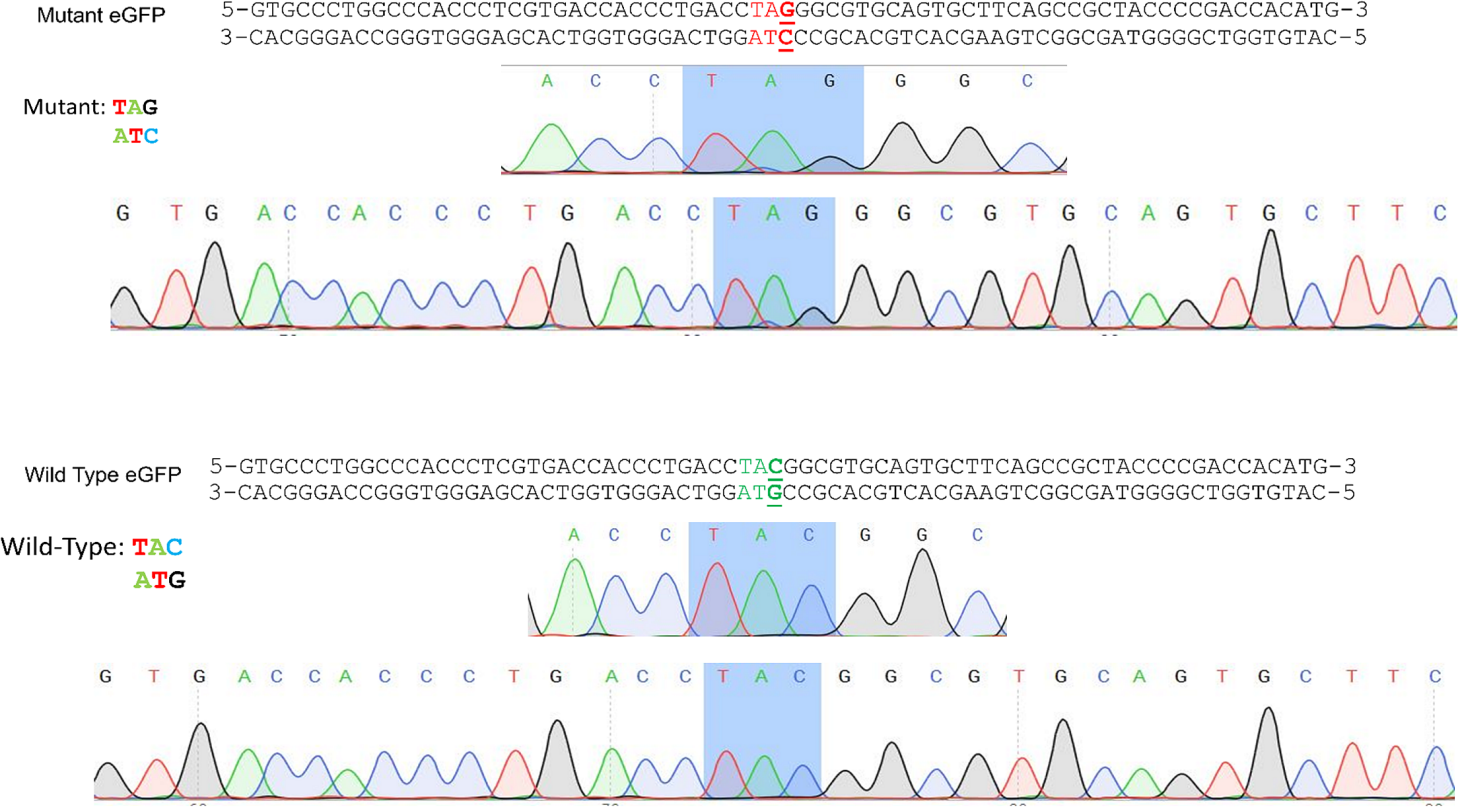
Sequence confirmation of ssODN/CRISPR edited cells. Unsynchronized HCT116-19 cells were electroporated under the following conditions; 2ug CRISPR 2C and 1.35ug 72NT at 5e5 cells/100ul. Cells were then sorted for GFP+ at 72 hours post electroporation. Immediately following cell sorting, DNA was isolated and the region surrounding the target base was amplified via PCR. Samples were submitted to Genewiz (South Plainfield, NJ) for sequencing analysis.

When we developed the mutant eGFP gene editing system for studying the mechanism of action, we defined gene correction frequency as the percent of eGFP^+^ cells within the entire viable population of cells that had been treated [1,2,5]. The rationale was that this frequency will more adequately represent the degree of correction presumably attainable in primary cells, where it will not be practical to (solely) select transfected cells to measure actual correction levels. There is however, an alternative for calculating gene editing frequencies based on the percent of cells that actually received the CRISPR/Cas9 construct. Thus, we provide, in Table 3, gene editing efficiencies adjusted to the level of transfection of the plasmid construct using different CRISPR, in the presence of the 72NT oligonucleotide. The transfection efficiency was based on the number of cells exhibiting fluorescence from the pX458 eGFP expression CRISPR construct. Here we present the normalized correction efficiencies for the three most active CRISPR/Cas9 complexes (vectors lacking the eGFP marker) that best catalyze gene editing. An obvious increase in correction frequency is observed even in unsynchronized cells, which, in the end, is the true target population type for in vivo human therapeutic application. To confirm gene editing at the DNA level, we isolated eGFP- positive cells by cell sorting and submitted the samples for direct DNA sequence analysis. A typical and reproducible sequence result is presented in Fig 9. The upper panel represents a DNA sequence from a control mutant eGFP gene while the lower panel represents the DNA sequence of a corrected cell from the sorted population. The TAG to TAC base conversion is readily observable to be complete and thus these data confirm and correlate the observed phenotype with genotypic analysis.

**Table 3:** Normalized Correction Efficiencies.

**CRISPR /Cas9**	**Dose**	**ssODN**	**Transfection (%)**	**Normalized Correction Efficiency (%)**	**SE**
pX458	2ug	-	67.92	-	1.28
pX458	2ug	1.35ug 72 PM	45.96	-	0.42
2c	2ug	1.35ug 72NT	45.96	6.81	0.39
3c	2ug	1.35ug 72NT	45.96	1.49	0.08
5c	2ug	1.35ug 72NT	45.96	4.04	0.07

Unsynchronized HCT116-19 cells were harvested and electroporated at a concentration of 5x10^5^cells/100ul with 2ug of the indicated CRISPR/Cas9 (2C, 3C, 5C and empty pX458 vector) plus 1.35ug of either 72NT or 72 PM. Following electroporation, transfection efficiency was determined after 24 hours of incubation by the percentage of total viable eGFP+ cells in the population. The normalized correction efficiency was determined after 48 hours of incubation as the percentage of total viable eGFP+ cells in the population divided by the transfection efficiency. Standard Error was calculated from two sets of data points generated over two separate experiments.

## Discussion

Single-stranded oligonucleotides and CRISPR/Cas9 complexes can be used in combination to direct nucleotide exchange in a precise and efficient fashion. We have used a mutant each eGFP gene as the target for gene editing reactions because it enables genotypic and phenotypic readout. The system provides a framework upon which mechanistic studies to define reaction parameters can be built. The introduction of an expression construct containing a CRISPR/Cas9 cleavage system and the appropriate single-stranded oligonucleotide leads to the correction of the mutant eGFP gene in the production of functional fluorescent protein. A series of CRISPR/Cas9 complexes that vary in position of cleavage, within the mutant eGFP gene, were found to produce a wide range of editing activities. Using an oligonucleotide that is complementary to the non-transcribed strand in the editing reaction affords a higher level of correction compared to its oligonucleotide counterpart hybridizing to the transcribed strand. Such strand bias has been identified in many previous reports [15,25–32]. Synchronization at the G1/S border with subsequent release produces an enriched population of cells undergoing DNA replication. This manipulation of the cell population produces a more amenable environment for gene editing activity [30,39–41]. The phenomenon of strand bias, however is complex and likely to be determined or influenced by the strand serving as the template for lagging strand synthesis rather than the transcription template. The data from early studies led to the fundamental model outlined in 2007 [2] expanded upon and reconfirmed in 2011 [3]. hese models are based on the foundational assumption that replication activity can modulate the mechanics of gene editing and the extent to which gene editing takes place when driven by single-stranded DNA. Lin et al [35] produced elegant data with CRISPRs suggesting that HDR is also occurring as the cells transit S-phase and possibly into G2, aligning with this notion.

Ran et al (2013) [42] showed that when single-stranded or double-stranded donor DNA was introduced into a targeted genomic site, with the objective of DNA insertion, no strand bias was observed. The objective of our experimental design is to fix or repair a single base mutation without the intended insertion of a donor fragment. Perhaps, our reaction is more dependent on DNA replication and a restriction on strand preference, as stated above. Different objectives using CRISPR and various donor DNA templates could take alternative routes and be governed by different reaction requirements. To this point and as an example, Davis and Maizels [43] reported that HDR is more active on the transcribed strand as compared to the non-transcribed strand in their system. These data were obtained from experiments in which the outcome of HDR reaction was measured after being initiated by a nick on one of the two DNA strands. There was no indication, however, as to which of these two strands served as a template for lagging strand synthesis, a key aspect of the strand bias phenomena we observe in our type of gene editing reactions. We see the same strand bias independent of DNA cleavage activity promoted by programmable nucleases.

CRISPR/Cas9 complexes that cleave at proximal positions relative to the target base are more efficient in promoting the reaction, directed by single-stranded oligonucleotides. This result aligns with recent data from our lab and others [15,44,45] then that suggest that the double strand break enables integration of the oligonucleotide more efficiently at the proper site. The use of modified CRISPR/Cas9 complexes, redesigned as single-strand endonucleases (Nickases), but at a level that is roughly 90% less than the wild type Cas9 enzyme for applications involving single nucleotide exchange. The combination of two single-stranded endonucleases (double nicking) does not recapitulate the level of gene editing activity seen with the intact complex. The activity from the combination of 2N and 5N reveal an interesting data set. Since these two Nickases act on the same DNA strand, it is possible that a gap is created that is large enough to hybridize productively to the single-stranded oligonucleotide. The single stranded character of such gapped DNA molecules can engage single-stranded DNA if the complementarity zone can be maintained for a certain period of time. By double nicking on the same strand, a long enough section of complimentary DNA may be available for productive annealing with the oligonucleotide. In general, however, a fully functional CRISPR/Cas9 complex able to cleave ds DNA is most productive in the execution of efficient gene editing reactions. The offset lengths shown in Fig 7 represent the distance in base pairs between the PAM-distal (5’) ends of the guide sequences of a pair of guide RNAs as defined by Ran et al. (2013) [38]. ollowing this convention, the enzymes used in this study range from +33bp to -96bp, producing nicks in a way which result in 3’ overhangs. It has been previously reported that only sgRNA pairs creating 5’ overhangs with offsets greater than -8bp between the guide sequences were able to mediate detectable indels [38], while we see detectable levels of correction, presumably as a result of indel formation.

The differing levels of gene editing activity promoted by CRISPR/Cas9 complex 2C versus 3C are striking. Both cleave the gene at the same site and yet nucleotide exchange promoted by 2C occurs at a fivefold higher level than nucleotide exchange promoted by 3C. In our study, 2C provided the highest level of activity while cleaving the DNA upstream (5’) from the target base. Since 3C cuts at the same site, the results were perplexing at first until one examines the hybridization potential of the guide RNA sequence of both 2C and 3C with 72 NT. The free energy of pairing of 3C with 72 NT was much lower than that of 2C. In fact, the entire guide sequence of 3C can be hybridized in perfect register with a section of 72 NT. CRISPR/Cas9 complexes 2C and 72 NT share the same polarity, thus they are unable to hybridize productively. This observation is reflected in the higher-level ΔG as seen in Fig 3. It is therefore quite likely that the single-stranded oligonucleotide,72NT, is titrating, at least in part, the guide RNA sequence of 3C, reducing the overall effectiveness of 3C in binding properly to the target site and promoting efficient DNA cleavage.

We tested this hypothesis directly and the data are presented in Fig 5A. The idea was that efficient cleavage activity of 2C and 3C should be easily identified by the loss of a restriction site in the DNA through the creation of a deleted DNA sequence. In this case, the recognition site for AvrII would be lost if the DNA had been modified at or near the cleavage site. If 72 NT was titrating the guide RNA of either 2C or 3C, it would reduce AvrII cleavage since CRISPR/Cas9 complex activity would have been inhibited by 72 NT. The results indicate that 72 NT inhibited only the cleavage activity of 3C, supporting our original hypothesis.

While the majority of CRISPR/Cas9 activity is currently directed toward gene knockout, when this genetic tool is used in combination with single-stranded oligonucleotides, to direct single nucleotide exchange, several additional reaction parameters need to be considered. When the objective is to functionally disable a gene, it may not be important as to where DNA cleavage takes place, although it is likely to be more effective at the 5’ end of the gene. In the case of gene editing however, where the aim is to enable single base repair, our data suggest that cleavage must take place at a proximal position relative to the mutant base. These data align completely with previously published work when the combination of TALENs and single-stranded DNA oligonucleotides was used to direct gene correction [15,44]. Consistent with many other reports, oligonucleotides that are complementary to the non-transcribed strand of the gene are more effective in promoting gene editing [25,26]. There are a number of different theories as to why such strand bias is observed [46–48]., the non-transcribed strand of the target gene is also the lagging strand in DNA replication thereby facilitating the incorporation of the oligonucleotide more easily into the growing replication fork [26]. When programmable nucleases are used to promote gene editing, the creation of an entry point, proximal to the target base, may provide a significant advantage in the gene editing reaction. Our results also point to an important difference in the use of TALENs versus RGEN technologies in gene editing. Since TALENs are composed of a series of binding domains coupled to a functional nuclease, the single-stranded oligonucleotide used to direct the nucleotide exchange will not affect TALEN cleavage nor TALEN activity since there is no guide RNA.

Based on the data presented in this report, we are beginning to define guidelines to assure efficient nucleotide exchange and gene correction. First, the oligonucleotide should be complementary to the non-transcribed strand of the gene. Second, the cleavage by the programmable nuclease should be within 20 to 50 bases, preferably upstream, of the nucleotide designated for change. Third, the nuclease system should be designed so that the guide RNA sequence is of the same polarity as the non-transcribed strand to avoid hybridization to the single-stranded oligonucleotide which, in most cases will be complementary to the non-transcribed strand. And fourth, double-stranded DNA cleavage is more efficient than single-stranded cleavage in providing an amenable target (entry point) for gene editing by single-stranded oligonucleotides.
